# Silver Nanoparticle Synthesis by *Rumex vesicarius* Extract and Its Applicability against Foodborne Pathogens

**DOI:** 10.3390/foods12091746

**Published:** 2023-04-23

**Authors:** Essam Mohamed Elsebaie, Nora Hamdy Mouhamed El-Wakeil, Azhar Mostafa Mohamed Khalil, Rasha M. Bahnasy, Galila Ali Asker, Marwa Fawzy El-Hassnin, Suzan S. Ibraheim, Marwa Fawzi Ahmed El-Farsy, Asmaa Antar Faramawy, Rowida Younis Essa, Mohamed Reda Badr

**Affiliations:** 1 Food Technology Department, Faculty of Agriculture, Kafrelsheikh University, Kafr El-Sheikh 33516, Egypt; 2 Nutrition &Food Science Department, Faculty of Home Economics, Al-Azhar University, Tanta 31512, Egypt; 3 Food Science &Technology Department, Faculty of Home Economics, Al-Azhar University, Tanta 31512, Egypt; 4 Food Science and Technology Department, Agriculture Faculty, Tanta University, Tanta 31512, Egypt

**Keywords:** foodborne pathogens, *Rumex* extract, AgNPs, cytotoxic activity, antibacterial activity

## Abstract

The consumption of foods polluted with different foodborne pathogens such as fungus, viruses, and bacteria is considered a serious cause of foodborne disease in both humans and animals. Multidrug-resistant foodborne pathogens (MRFP) cause morbidity, death, and substantial economic loss, as well as prolonged hospitalization. This study reports on the use of aqueous *Rumex* leaf extract (ARLE) in the synthesis of silver nanoparticles (ARLE-AgNPs) with versatile biological activities. The synthesized ARLE-AgNPs had spherical shapes with smooth surfaces and an average hydrodynamic size of 27 nm. ARLE-AgNPs inhibited the growth of *Escherichia coli* ATCC25721, *Pseudomonas aeruginosa* ATCC27843, *Streptococcus gordonii* ATCC49716, *Enterococcus faecalis* ATCC700813, and *Staphylococcus aureus* ATCC4342. The ARLE-AgNPs were more active against *Escherichia coli* ATCC25721 than other harmful bacterial strains (26 ± 3 mm). The zone of inhibition for antibacterial activity ranged between 18 ± 3 mm and 26 ± 3 mm in diameter. The nanoparticles’ MIC values varied from 5.19 µg/mL to 61 µg/mL, while their MBC values ranged from 46 µg/mL to 119 µg/mL. The nanoparticles that were created had antioxidant potential. The cytotoxic activity was tested using normal fibroblast cell lines (L-929), and the enhanced IC50 value (764.3 ± 3.9 g/mL) demonstrated good biological compatibility. These nanoparticles could be evolved into new antibacterial compounds for MRFP prevention.

## 1. Introduction

Nowadays, the spoilage of foods is a major issue everywhere in the world because of the presence of foodborne pathogens [1,2]. Additionally, the public’s health is now seriously threatened by the emergence of new harmful bacterial strains that are resistant to antibiotics already in use, necessitating the urgent development of a powerful new generation of bactericides [3]. Because of the high expenses associated with treatment, hospitalization, and epidemiological inquiry, infections brought on by foodborne microbes are regarded as a serious problem in worldwide public health [4]. The World Health Organization estimates that there are 600 million instances of foodborne infections each year, resulting in 420,000 deaths per year, or 1 in 10 individuals becoming sick from eating contaminated food [5]. In recent years, foodborne pathogens, particularly bacteria with multidrug resistance (MDRS), have posed significant hurdles for therapeutic agents and have been growing rapidly all over the world [6]. A variety of causes, such as incorrect antibiotic prescription and sales, the usage of antibiotics outside the healthcare industry, and the inherent genetic aspects of bacteria, have all contributed to the rise of bacteria with MDRS [7]. It is very important to consider food safety, quality, and ways to prolong its shelf life by discovering novel antimicrobials and antioxidant substances because food is one of the most essential elements for all living organisms. In recent years, nanotechnology has become a new and promising field for the production of nanomaterials, or nanoparticles with a diameter of less than one nanometer, which, because of their large surface areas relative to their volumes and special chemical and physical characteristics, have antimicrobial effects [8]. Silver nanoparticles (AgNPs) have been extensively employed among the various metal nanoparticles because of their powerful antibacterial [8], antiproliferative [9], and antifungal actions [10]. AgNPs have a lot of advantages that make them effective antibacterial agents. They have excellent antiparasitic and antipathogenic abilities against a wide variety of pathogenic microorganisms, and they have a low toxicity to humans [11]. AgNPs have been widely employed in biofertilizers, food preservation, medications, cosmetics, and food packaging because of their strong antibacterial qualities [12]. Because several types of bacteria have the highest sensitivity to Ag, Ag-based compounds are advantageous for antimicrobial applications against foodborne infections as well as antioxidants to preserve food quality.

AgNPs have a cytotoxic effect against mammalian cells; scientists studied the cytotoxic effect of uncoated AgNPs and the AgNPs coated with either chemical or biological substances [13]. The factors contributing to the cytotoxicity of AgNPs are the shape, size, and surface charge or coating and the release of the ionic form of silver. AgNPs may disrupt the membrane of the cell, affect the production of ATP and the replication of DNA, change the gene expression, and oxidize the biological compartments of the cell through the production of ROS. The silver ions released by AgNPs may block the respiratory chain of the microorganisms in the cytochrome oxidase and NADH–succinate dehydrogenase region [14].

For the production of AgNPs, both chemical and physical methods are often used [15]. Despite the ability of physical and chemical methods to synthesize nanoparticles of a particular size and shape, the use of hazardous materials (toxic reducing agents, potentially toxic surfactants, or capping agents to control the size of NPs) and low economic feasibility make their application limited [16]. Commonly used chemical and physical methods are chemical reduction, ion sputtering, sol gel, etc., which have higher energy requirements and include improvident purifications [17,18]. Simple and environmentally friendly approaches should be used instead of these costly and hazardous ones because they are more effective. The green synthetic nanoparticles approach has various benefits over the chemical and physical methods since it is economical, ecofriendly, and extremely easy to handle in commercial-scale operations without utilizing a lot of hazardous chemicals, high temperatures, high pressures, or excessive energies [19,20,21].

A very large number of papers have used plant resources in the green fabrication of AgNPs. The use of plant extracts has recently emerged as a superb option for the fabrication of AgNPs. This green technique is more advantageous since it employs plant-based flavonoids, enzymes, proteins, and so on, which are devoid of hazardous chemicals and naturally maintain a consistent chemical composition [22,23].

*Rumex vesicarius* is an edible wild plant known as “Humaidah” in Arabic and “Bladder Dock” in English that is harvested in the spring and consumed fresh or cooked [24]. There are numerous significant medical applications for *Rumex vesicarius*, including the treatment of tumors, diuretics, hepatic illnesses, astringents, poor digestion, purgatives, constipation, antispasmodics, calculi, stomachics, heart problems, tonics, pains, laxatives, diseases of the spleen, appetizers, hiccoughs, analgesics, flatulence, asthma, and as an antibacterial agent [25].

*Rumex* is a member of the *Polygonaceae* and is known to produce a variety of biologically significant secondary metabolites, including phenolic acids, stilbenoids, leucoanthocyanidins, flavonoid glycosides, anthraquinones, and steroids [26,27,28]. There has not yet been any information published on the fabrication of AgNPs using aqueous *Rumex* leaf extracts (ARLE). Therefore, the goal of the current work was to generate ARLE-AgNPs (AgNPs generated using ARLE) and evaluate their bioactive characteristics, including their ability to operate as an antibacterial agent versus MRFP and their antioxidant capacity. Additionally, the L-929 cell line cytotoxicity test was evaluated.

## 2. Materials and Methods

### 2.1. Materials

All the chemicals and media used in the study were of an analytical grade, purchased from Sigma–Aldrich (Mumbai, India). Fresh Egyptian *Rumex* (Humaidah) leaves (RLs) were gathered from the faculty farm at the University of Kafrelshiekh, Egypt, in April 2019. The RLs were cleaned with water from the tap, followed by distilled water, left to dry inside the shade, and then crushed into a fine powder (60 mesh) with the use of a grinder. All microbial strains were obtained from the Microbiology Research Laboratory at Kafrelshiekh University.

### 2.2. Preparation of ARLE

Six grams of RL powder was combined with 60 mL of sterile, distilled water to create an ARLE, which was then sonicated for 15 min. Repeated centrifugation was used to purify the sonicated ARLE. The pure ARLE was filtered using No. 1 Whatman filter paper, and the filtrate was kept at 4 °C for future use [29]. The filtered ARLE (pH 7.5) was employed within three hours to generate Ag NPs.

### 2.3. Analysis of the ARLE

Total Phenolic content was determined in the ARLE using the Folin–Ciocalteu colorimetric method [30] and the obtained results were explicated as mg gallic acid equivalent (GAE)/g extract. Chromatographic analyses were carried out in an HPLC (Shimadzu LC-10A; Kyoto, Japan) unit, as described by Elsebaie and Essa [31]. The final data were calculated as a percentage of each component from the total phenolic components.

### 2.4. AgNPs’ Biosynthesis

The ARLE was mixed in a 1:10 mL ratio with a 6 mM AgNO3 solution and stirred at room temperature at 200 rpm until the color turned brown as an indicator of the AgNPs’ formation. The supernatant was then removed, and the sediment pellets were obtained by centrifuging the brown solution at 10,000 rpm for twenty minutes. After that, the pellets were dried at 60 °C for twelve hours in an air oven and ground down. Finally, the powder was autoclaved and kept at 4 °C [32,33].

### 2.5. Characterization of ARLE-AgNPs

UV-Vis spectroscopy (Shimadzu 2450UV/Vis, Kyoto, Japan) was used to record UV-vis spectra in the wavelength range of 200 to 700 nm at room temperature. Quartz cuvettes were used for the analyses. From 0 to 180 min, the reaction mixture was measured spectrophotometrically every 30 min [34]. For the blank solution, distilled water was used. Freeze-dried ARLE-AgNPs (0.2 mg/mL) were dispersed in double distilled water by short gentle sonication. Dynamic scattering light analysis using a particle size analyzer (PSA, Malvern Nano-ZS 2000, Leeds, UK) was done to indicate the size of the ARLE-AgNPs generated in triplicates on undiluted samples at 25 °C following the approach given by Khan et al. [35]; while for the zeta-potential measurement, the ARLE-AgNPs were dispersed in an aqueous solution and the measurements were performed on undiluted samples at 25 °C using Zeta-Nano ZS2000. Zeta-potential values were measured in triplicates according to the Smoluchowski equation.

SEM analysis was performed with a scanning electron microscope (JSM-7001F, Osaka, Japan) to examine the morphological structure of the ARLE-AgNPs as reported before by Ramamurthy et al. [36]. The energy dispersive x-ray spectroscopy (EDX) method was used to determine the elemental composition, purity, and relative abundance of the green synthesized ARLE-AgNPs [37].

### 2.6. Antibacterial Activity of ARLE-AgNPs

#### 2.6.1. Bacterial Inoculums Preparation

MRFP, including *Escherichia coli* ATCC25721, *Pseudomonas aeruginosa* ATCC27843, *Streptococcus gordonii* ATCC49716, *Enterococcus faecalis* ATCC700813, and *Staphylococcus aureus* ATCC4342, were cultured and incubated for 18 h at 37 °C in a nutritional broth medium. The culture’s optical density was kept at 1.0 for use in subsequent investigations, and the growth was observed at 600 nm by a UV/Vis spectrophotometer [38].

#### 2.6.2. Antimicrobial Activity Measurement

For determining the antibacterial activity of ARLE-AgNPs, the disc diffusion technique was applied. In sterilised conical flasks, 15 mL of nutrient agar was poured, infected with 0.2 mL of bacterial culture suspension, gently stirred, then put into sterilised petri plates and left to harden. The ARLE-AgNPs weighed 0.5 g and were dissolved in 10 milliliters of 5% dimethyl sulfoxide. The discs were immersed in the ARLE-AgNPs solution. Before placing the discs in agar media with the microbial cultures, they were air dried in an aseptic condition. After 24 h of incubation at 37 °C, the inhibition zones were measured in millimeters [39]. Furthermore, the antibacterial activity of the ARLE-AgNPs on the bacterial strains was confirmed using the microbroth dilution technique, as well as the determination of the minimum inhibitory concentration (MIC) and minimum bactericidal concentration (MBC) [40]. The MIC and MBC of the specimen were measured using the broth dilution technique, as previously outlined by Kubo et al. [41]. Different dilutions of the ARLE-AgNPs (100–1.25%) were used. The initial serial dilutions of the ARLE-AgNPs in nutritional broth containing nanoparticles were carried out continuously until the dilution reached 1.25%. Only nutritional broth was used in the control vial. This experiment was carried out on all the chosen bacteria strains. The bacteria were injected, and the MIC was defined as the lowest concentration that prevented bacterial growth at the lowest dosages. After that, the sample was placed on nutrient agar tubes and incubated for 24 h at 37 °C to calculate the colony-forming unit (CFU) value. The data for the MIC and MBC were given as µg/mL.

### 2.7. Synergistic Activity of ARLE-AgNPs

The disc diffusion method previously described by Rahim and Mohamed [42] was used to explore the synergetic effect of the ARLE-AgNPs and the ampicillin antibiotic. To detect the synergistic action of the ARLE-AgNPs and the ampicillin antibiotic, sterile filter paper discs (6 mm in diameter) were injected with 10 g/disk of the antibiotic ampicillin, 10 g/disk of the ARLE-AgNPs, and 10 g/disk of both the antibiotic ampicillin and the ARLE-AgNPs. The soaked discs were then placed on top of the bacterial culture dishes. The plates were then incubated for 24 h at 25 °C. A Vernier caliper was used to measure the inhibitory zone widths, and the synergistic efficacy of the ARLE-AgNPs and ampicillin antibiotic was calculated [43].

### 2.8. Antioxidant Activity of ARLE-AgNPs

#### 2.8.1. DPPH Scavenging Activity

The 1-1-diphenyl-2-picryl-hydrazyl (DPPH) technique was used to assess the free radical scavenging activity. DPPH was produced as a 0.1 mM solution in methanol. 3 mL of ARLE and ARLE-AgNPs in various concentrations (ranging from 10 to 50 mg/Ml) were mixed with 1 mL of DPPH solution. UV/Vis spectrophotometer (Shimadzu 2450UV/Vis, Japan) was used to detect absorbance at 517 nm after 30 min of incubation [44]. The equation listed below will be used to determine the % scavenging activity values:

(1) $$\mathrm{DPPH}\;\mathrm{Scavenging}\;\%=\left[\;\frac{Blank\;absorbance-Sample\;absorbance\;}{Blank\;absorbance}\right]\times 100$$

#### 2.8.2. Nitric Oxide (NO) Radical Scavenging Activity

The modified techniques of Sousa et al. [45], which are based on the detection of NO radicals produced from sodium nitroprusside by the Griess reaction, were used to determine the NO radical scavenging activity. To produce NO radicals, 200 mL of 20 mM sodium nitroprusside was reacted for 90 min at room temperature with 200 mL of the ARLE-AgNPs and ARLE at concentrations in the same range. At 540 nm, the optical density was then determined. The equation listed below will be used to determine the % scavenging activity values:

(2) $$\mathrm{NO}\;\mathrm{radical}\;\mathrm{Scavenging}\;\%=\left[\;\frac{Blank\;absorbance-Sample\;absorbance\;}{Blank\;absorbance}\right]\times 100$$

#### 2.8.3. Hydrogen Peroxide Scavenging Assay

The hydrogen peroxide test was used to measure the free radical scavenging activity. A 40 mM hydrogen peroxide solution in 0.1 M phosphate buffered saline (pH 7.4) was created. A 600 μL solution of H_2_O_2_ will be quickly mixed with 1 mL of samples containing ARLE and ARLE-AgNPs at various concentrations (ranging from 10 to 50 mg/ML). After 10 min of incubation at room temperature against a blank (without hydrogen peroxide), the absorbance was measured at 230 nm in a UV/Vis spectrophotometer (Shimadzu 2450UV/Vis, Japan). The equation that follows will be used to determine the percentage of hydrogen peroxide scavenging. Quercetin has been utilised as a positive control sample [46].

(3) $$\mathrm{Radical}\;\mathrm{Scavenging}\;\%=\left[\;\frac{Blank\;absorbance-Sample\;absorbance\;}{Blank\;absorbance}\right]\times 100$$

### 2.9. In Vitro Biocompatibility Assay

The biocompatibility of the ARLE-AgNPs was assessed by determining the percentage of cell viability after treating the L-929 normal cell lines with the ARLE-AgNPs. The L-929 cells were cultured for 24 h at 37 °C (5% CO_2_) in flasks containing 10% fetal bovine serum, M-199 medium, and Dulbecco’s Modified Eagle’s Medium (DMEM). After the incubation time, the associated cells were centrifuged after being trypsinized for 3–5 min to separate the individual cells (800 rpm, 10 min). The cells were counted and dispersed on a 96-well enzyme-linked immunosorbent assay (ELISA) plate with 5000 cells in each well for 24 h to produce a monolayer with a 70–80% confluence. The ARLE-AgNPs have the ability to significantly lower the amount of ATP present in the cell, which eventually results in mitochondrial damage and raises the formation of reactive oxygen species (ROS) in a dose-dependent way [47]. As a result, the toxicity of the AgNPs after 5 h was assessed in triplicate at various concentrations between 100 and 1000 mg/mL. Each well received 200 mL of a 3-(4,5-dimethylthiazol-2-yl)-2,5-diphenyltetrazolium bromide (MTT) solution, which was added to determine the vitality of the cells (4–5 h).

### 2.10. Statistical Analysis

All treatments and analyses were performed in triplicate. All the data were presented as mean ± standard deviation (SD). Using SPSS software (version 16.0 for Windows, SPSS Inc., Chicago, IL, USA), the data were analyzed for variance (ANOVA) and the Duncan test was used to determine the significant (*p* < 0.05) amongst the treatments.

## 3. Results and Discussion

### 3.1. Identification of ARLE Polyphenolic Compounds

The ARLE total phenolic content was 26.13 mg GAE/g extract. The composition of polyphenolic compounds extracted from the glasswort air part were determined by high performance liquid chromatography (HPLC) and the results are listed in Table 1. It should be noted that the ARLE contains 12 phenolic compounds. The most predominant phenolic compounds presented and identified in the ARLE were Epicatechin gallate (27.42%), Pyrogallol (23.32%), Catechin (11.45%), and β-OH Benzoic (10.80%). Meanwhile, caffeic, Ferulic, Gallic, and Vanillic were the least present phenolic components in the extract. The obtained results were in line with those obtained by Laouini and Ouahrani [48], Mohammed et al. [49], and Workineh [50].

### 3.2. Characterization of ARLE-AgNPs

**Figure 1:**
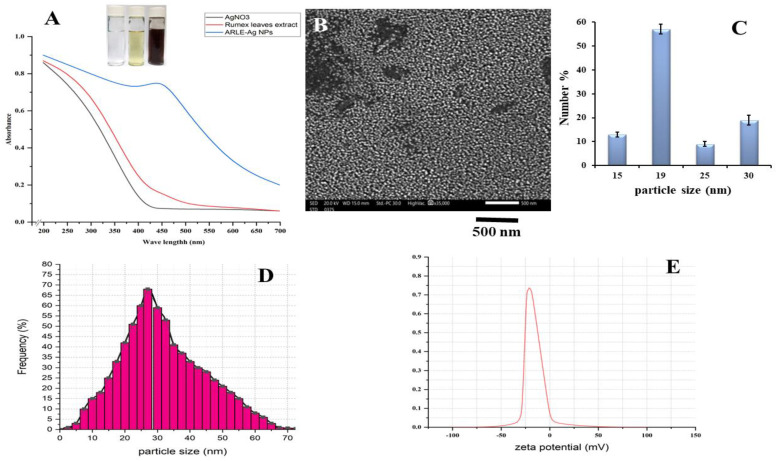
Physico-chemical characteristics of the ARLE-AgNPs: (**A**) ARLE-AgNPs UV/Vis absorbance spectrum, (**B**) SEM image, (**C**) SEM particle size histogram, (**D**) hydrodynamic particle size distribution by DLS, (**E**) the ARLE-AgNPs surface charge and the zeta potential value.

Upon addition of the ARLE, the color of the silver nitrate solution changed to dark brown after 6 h of incubation, indicating the formation of silver nanoparticles. The change in color by the extract demonstrates the reduced ability of the plant extract for the synthesis of the AgNPs [51]. The brownish color appears due to the coherent oscillation of conduction band electrons at the nanoparticle’s surface, resulting in surface plasmon resonance (SPR) [52]. The UV/VIS spectrum of the ARLE-AgNPs produced is shown in Figure 1A. The AgNO_3_ solution and the ARLE-AgNPs were incubated, and the result was the formation of a dark-brown colloidal suspension that displayed the distinctive SPR band in the visible spectrum. For pure ARLE and neat AgNO_3_ solutions, no distinguishable peaks were seen. Additionally, none of the colloidal solutions created showed an obvious longitudinal SPR peak, suggesting that the produced nanoparticles had an isotropic shape.

The ARLE contains polyphenolic compounds that work as reducing factors by giving electrons to metal ions. One of the most prevalent phenolic compounds detected in the *Rumex* species is epigallocatechin gallate. The structure of epigallocatechin gallate has two rings with identical local structures; the first ring is the (D-ring) with the gallate group and the second ring is the (B-ring) with a pyrogallol-type structure, so the reaction is defined as a single ring because both rings include three OH-groups that could potentially participate in the ARLE-AgNPs’ formation [53].

The SEM picture of the ARLE-AgNPs is displayed in Figure 1B. According to the SEM pictures, the majority of silver nanoparticles have spherical shapes with smooth surfaces, are evenly distributed, and are arranged in close, compact clusters.

The SEM micrograph suggests that the particles have an average diameter of 19 nm (Figure 1C). The hydrodynamic size of the ARLE-AgNPs was investigated using the dynamic light scattering (DLS) method while the surface charge of the nanomaterials was investigated using the zeta potential analysis technique [54]. The ARLE-AgNPs revealed hydrodynamic size in a range of >5 to >70 with an average of 27 ± 0.21 nm with a polydispersity index of 0.564 ± 0.07, as demonstrated in Figure 1D. The particle size of the silver nanoparticles detected by DLS was larger than that estimated by the SEM examination, which could be due to the accumulation of extra hydrate layers on the surface of the AgNPs [55].

The dimension values exhibit a strong correlation when the average size of the ARLE-AgNPs calculated using SEM and DLS are compared. The small diameter of the synthesized silver nanomaterials revealed the high effectiveness in utilizing the green approach for the synthesis of the AgNPs [56]. SEM analysis of the AgNPs synthesized using several plant extracts were predominantly spherical shaped [57,58,59,60]. The level of stability of colloidal dispersion nanoparticles is described by the zeta potential. The great stability of the ARLE-AgNPs is shown by their zeta potential, which was −30 ± 3.46 mV when they were produced. According to Figure 1E, a negative value indicates that the nanoparticles’ surfaces are negatively charged, which creates a strong electrostatic repulsion force between the particles, contributing to their stability in aqueous solutions [61]. This enhances the stability of the formulation of AgNPs without the usage of a capping agent. For use in a therapeutic proposal, this is crucial.

**Figure 2:**
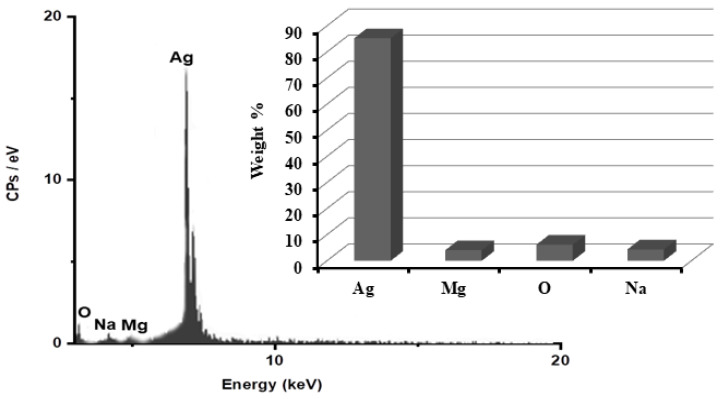
Identification of the ARLE-AgNPs’ elemental composition.

In order to determine the purity of the AgNPs and their whole chemical composition, an energy dispersive X-ray spectroscopy (EDX) examination of the reduced ARLE-AgNPs was carried out. According to the EDX spectrum, the proportion of Ag metal was substantial in contrast to other chemical components, as shown in Figure 2. Silver (Ag) was 85.3%, oxygen (O) 6.2%, Na 4.4%, and Mg 4.1%, respectively. The bioactive compounds from the plant extracts that are attached to the surface of the biosynthesized NPs may have been the source of additional elements [62].

### 3.3. ARLE-AgNPs’ Antimicrobial Activity

The antibacterial effect of AgNPs is attributed to at least one of their capacities to anchor to the cell wall, generate pits and modify cell membrane permeability leading to cell death [63], their production of free radicals that induce permeability in the cell membrane, their deactivation of enzymes by sulfhydryl groups blocking, and their suppression of respiration enzymes by Ag ions resulting in the release of reactive oxygen species. Furthermore, the soft acidic action of Ag with soft base Sulfur and phosphorus can cause DNA to be destroyed [64], and they can also stop bacteria from growing by preventing them from transmitting signals [65], which is done by dephosphorylating peptide substrates upon tyrosine residues. Major public health issues are caused by both Gram-positive (+) and Gram-negative (+) bacterial strains, and they are made much worse by the introduction of strains that are MDRS [8]. The ARLE-AgNPs were tested for antibacterial activity against Gram-negative (*Escherichia coli ATCC25721* and *Pseudomonas aeruginosa ATCC27843*) and Gram-positive (*Streptococcus gordonii ATCC49716, Enterococcus faecalis ATCC700813,* and *Staphylococcus aureus ATCC4342*) pathogens using the agar disc diffusion technique. 

Table 2 displays the zones of inhibition (mm) surrounding each disc containing the ARLE-AgNPs. All the examined microorganisms had a zone of inhibition that was between 18 ± 3 and 26 ± 3 mm in diameter. Among the examined bacteria, *Enterococcus faecalis ATCC700813* was determined to be the most resistant, with a minimal inhibitory zone of 16 ± 2 mm. *Escherichia coli ATCC25721* had the greatest inhibition zone of 26 ± 3 mm. It was discovered that the ARLE-AgNPs worked well as an antibacterial agent against the tested microorganisms. Table 1 shows the MIC values at which no apparent growth of the tested microorganisms was observed. It was discovered that the MIC values for the ARLE-AgNPs against all the microorganisms under investigation ranged from 5.19 g/mL to 61 g/mL. Among the studied bacteria, *Escherichia coli ATCC25721* were proven to be the most susceptible with a MIC value of 5.19 μg/mL, whilst *Enterococcus faecalis ATCC700813* was confirmed to be extremely resistant with a MIC value of 61 μg/mL. The MBC is the smallest amount of any antibacterial ingredient that kills all the bacterial community (100%) and does not exhibit any possible growth when spread on agar media. Table 2 displays the MBC of the tested organisms for the ARLE-AgNPs. The values of MBC for all the microorganisms under investigation were observed to vary from 46 µg/mL to 119 µg/mL. Among the studied bacteria, *Escherichia coli ATCC25721* was shown to be the most susceptible with an MBC value of 46 g/mL, whilst *Enterococcus faecalis ATCC700813* was reported to be very resistant with an MBC value of 119 g/mL.

Results in Table 2 show that the ARLE-AgNPs have a lower impact on Gram-positive bacterial growth than they do on Gram-negative bacterial growth. This is because Gram-positive and Gram-negative bacteria have different cell walls in terms of their structural composition. The Gram-negative bacteria have a lipopolysaccharide membrane on the outside, followed by a narrow (7–8 nm) layer of peptidoglycan on the inside [66]. Despite the fact that lipopolysaccharides are made up of covalently bonded lipids and polysaccharides, they possess strength and stiffness.

The weak positive charge present on the AgNPs is drawn toward the lipopolysaccharide negative charges [67]. However, zeta potential measurements in the current research, as seen in Figure 1, indicated the existence of negatively charged ARLE-AgNPs. According to some, these negatively charged ARLE-AgNPs can kill Gram-negative bacteria through metal depletion [68]. In contrast, the Gram-positive bacteria’s cell wall is mostly made of a dense layer (between 20 and 80 nm) of peptidoglycan, which is a tri-dimensional stiff structure made of linear polysaccharide chains that are cross-linked via short peptides [69]. The cell membranes have fewer AgNP attachment sites because of their stiffness and extensive cross-linking, and they are also more difficult to penetrate.

The green synthesized silver nanoparticles have been shown to have antibacterial activity against multi-drug bacterial strains [70]. The obtained results were in line with previous studies which found that nanoparticles that have been synthesized using a floral extract of *Chrysanthemum indicum* L. [71], *Acorus calamus* extract [33], rhizome extract of *Curcuma longa* and *Zingiber officinale* [72], and *Moringa oleifera* flower [73] have a high antioxidant and antimicrobial properties.

### 3.4. Synergistic Activity of ARLE-AgNPs

Table 3 displays the width of the inhibitory zone (mm) around antibiotic discs containing and not containing the ARLE-AgNPs and ARLE discs versus the tested microbial strains. The results showed that the inhibition zones in the case of using the ARLE were lower than using the antibiotic discs containing and not containing the ARLE-AgNPs. Ampicillin’s antibacterial activity versus the tested microorganisms was enhanced when the ARLE-AgNPs were present. *Pseudomonas aeruginosa ATCC27843* had the largest percentage of fold growth inhibition, followed by *Escherichia coli ATCC25721, Staphylococcus aureus ATCC4342, Streptococcus gordonii ATCC49716,* and *Enterococcus faecalis ATCC700813*. The bonding process between the antibiotic and the AgNPs might be what is increasing the synergistic impact. Numerous active groups, including hydroxyl and amido groups, are present in antibiotic compounds, which readily chelate with AgNPs. Recent studies by Batarseh [74] have demonstrated that the bactericidal action was reliant on silver (I) chelating, which hinders the unwinding of DNA. Ampicillin works by lysing cell walls, and the particles of the drug can connect with one another through van der Waals forces as well as other weakening bonds. In the end, the antimicrobial groups are brought into direct contact with the AgNPs, in which the ampicillin monomers encircle the nanosilver core. By acting on the cell wall, the ampicillin molecules cause cell wall lysis, which promotes the diffusion of the AgNPs into the bacteria. The DNA-interacting AgNP-ampicillin complex stops DNA unwinding, which causes more severe harm to bacterial cells [74]. As a result, in this work, biosynthesis of ARLE-AgNPs is recognized to be inexpensive, efficient, environmentally friendly, and effortless.

### 3.5. Antioxidant Activity of ARLE-AgNPs

Metal nanoparticles have strong antioxidant properties [75]. It is well known that biogenic AgNPs have a larger capacity for donating H^+^ ions, which leads to a decrease in non-reactive substances [76]. H_2_O_2_ has a role in the cellular energy system [77]. In the current study, we evaluated ARLE and ARLE-AgNPs’ capacity to scavenge radicals. This was discovered to be dose-dependent, which is consistent with a recent finding [77].

**Figure 3:**
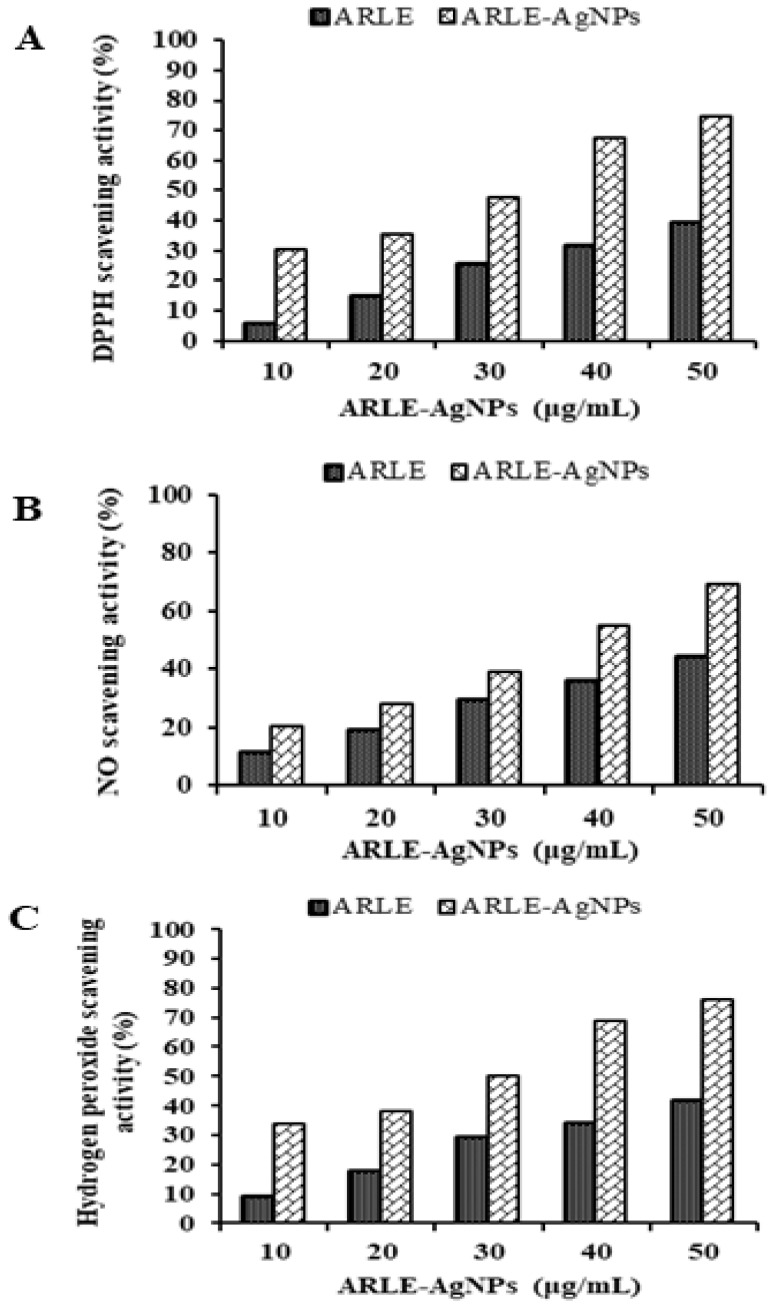
Radical scavenging activity of ARLE-AgNPs and ARLE: (**A**) DPPH free radical, (**B**) Nitric oxide free radical, and (**C**) Hydrogen peroxide free radical.

The ARLE-AgNPs and ARLE had significantly different DPPH, NO, and H_2_O_2_ radical scavenging activities, which were found to be maximal in the ARLE-AgNPs at a concentration of 50 μg/mL for DPPH (74.52%), NO (69.09%), and H_2_O_2_ (76.44%), and minimal in the ARLE at the same concentration of 50 μg/mL for DPPH (39.18%). As shown by the results in Figure 3, the ARLE-AgNPs were capable of performing strong antioxidant activities by giving H+ ions, which lead to the decrease of non-reactive species versus oxidizing agents. It is possible that the strong antioxidant activity was brought on by the capping of antioxidant components such as terpenoids, phenolic compounds, genipin, and crocin from ARLE to ARLE-AgNPs, as previously found by Muniyappan and Nagarajan [78] for Dalbergia spinosa leaves-AgNPs. Several previous investigations using silver nanoparticles reached a similar conclusion [9,22,43,44]. The effectiveness of ARLE-AgNPs in the context of modern drug development can be viewed as a source of an alternate antioxidant for lowering free radicals.

### 3.6. Biocompatibility Study

**Figure 4:**
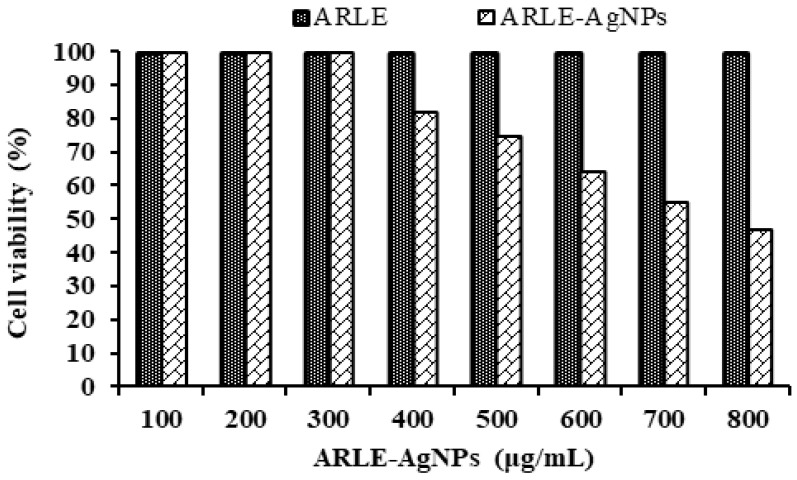
Biocompatibility activity analysis of ARLE-AgNPs and ARLE on normal L929 fibroblast cell lines after 5 h.

Understanding the biocompatibility of ARLE-AgNPs is critical for their effective application in biomedicine and their direct usage by humans as food additives. To assess their biocompatibility, the cytotoxicity of ARLE-AgNPs was also evaluated against normal L-929 fibroblast cells. A key worry is the toxicity of AgNPs when used in biological applications, as well as their safety. The L-929 cell line was not inhibited by the ARLE-AgNPs in the current investigation when used at lower doses (up to 300 µg/mL). As the concentration of the ARLE-AgNPs rose, the normal L-929 cell viability percentages decreased (Figure 4).

The ARLE-AgNPs’ IC50 value for the typical L-929 cell lines was determined to be 721.33 µg/mL. ARLE-AgNPs are very biologically compatible and may be used safely in the human body, according to the IC50 value. The ARLE showed its potential utility in the manufacture of ARLE-AgNPs by demonstrating no toxicity towards the L-929 cell line. Our findings are in line with those obtained by Lin, et al. [79], Amooaghaie, et al. [80], Rao, et al. [81], and Govindappa, et al. [82].

## 4. Conclusions

The Ag NPs emerged as the preferred option, especially when combined with plant extracts, due to their widespread availability, low cost, lack of toxicity, and environmentally friendly nature. The shape of the synthesized ARLE-AgNPs was found to be nearly spherical with an average hydrodynamic diameter of 27 nm. The ARLE-AgNPs created in this work showed strong antibacterial and antioxidant activity. The ARLE-AgNPs inhibited the growth of *Escherichia coli ATCC25721*, *Pseudomonas aeruginosa ATCC27843*, *Streptococcus gordonii ATCC49716*, *Enterococcus faecalis ATCC700813*, and *Staphylococcus aureus ATCC4342*. The synthesized AgNPs showed greater efficiency for gram-negative bacteria. The synergistic antibacterial effect with the combination of nanosilver and ampicillin has more potential, when compared with other antibiotics. The ARLE-AgNPs created in this work showed strong antibacterial and antioxidant activity. Furthermore, it was demonstrated that the ARLE-AgNPs were more effective than the ARLE at inducing cytotoxicity in L-929 cells. ARLE-AgNPs may be used to create a wide range of products, such as food packaging materials, coatings for medical equipment, and antimicrobial medications.

## Figures and Tables

**Table 1:** Polyphenolic compounds % in ARLE.

Phenolic Compound	% of the Total	Phenolic Compound	% of the Total
Gallic	2.80	caffeic	1.84
Protocatechuic	5.19	Vanillic	3.93
Pyrogallol	23.32	Caffeine	2.60
Epicatechin gallate	27.42	Ferulic	1.61
Chlorogenic	4.09	Β-OH Benzoic	10.80
Catechin	11.45	Epi-catechin	4.95

**Table 2:** Antibacterial activity of ARLE-AgNPs.

Bacteria	Inhibition Zones (mm)	MIC (µg/mL)	MBC (µg/mL)
**Gram-negative bacteria**			
*Escherichia coli* ATCC25721	26 ± 3 ^a^	5.19 ^e^	46 ^e^
*Pseudomonas aeruginosa* ATCC27843	22 ± 2 ^b^	14.07 ^d^	61 ^d^
**Gram-positive bacteria**			
*Streptococcus gordonii* ATCC49716	18 ± 3 ^d^	46.41 ^b^	92 ^c^
*Enterococcus faecalis* ATCC700813	16 ± 2 ^e^	61.00 ^a^	101 ^b^
*Staphylococcus aureus* ATCC4342	20 ± 2 ^c^	40.13 ^c^	119 ^a^

Data are presented as mean ± SD. Means with different superscripts lowercase letters in a column are significantly different at *p* < 0.05.

**Table 3:** Inhibition zones (mm) of antibiotics (with and without ARLE-AgNPs) against gram-positive and gram-negative bacteria.

Bacteria	Inhibition Zones (mm)			Fold Increase % = [(b − a)/a] × 100
	Ampicillin (a)	ARLE	Ampicillin + ARLE-AgNPs (b)	
*Escherichia coli* ATCC25721	19	10	32	68.42
*Pseudomonas aeruginosa* ATCC27843	15	5	26	73.33
*Streptococcus gordonii* ATCC49716	16	7	21	31.25
*Enterococcus faecalis* ATCC700813	14	5	18	28.57
*Staphylococcus aureus* ATCC4342	18	8	25	38.89
Overall synergistic antibacterial effect (%) = 48.09				

## Data Availability

The authors confirm that the data supporting the findings of this study are available within the article.
